# Editorial: Women in science: Pulmonary rehabilitation

**DOI:** 10.3389/fresc.2023.1130757

**Published:** 2023-02-09

**Authors:** Jana De Brandt, Ana Oliveira, Cristina Jácome

**Affiliations:** ^1^ Faculty of Medicine, Department of Community Medicine and Rehabilitation, Section of Physiotherapy, Umeå University, Umeå, Sweden; ^2^ Lab 3R – Respiratory Research and Rehabilitation Laboratory, School of Health Sciences (ESSUA) and Institute of Biomedicine (iBiMED), University of Aveiro, Aveiro, Portugal; ^3^ School of Rehabilitation Science, McMaster University, Hamilton, ON, Canada; ^4^ Department of Community Medicine, Information and Health Decision Sciences, CINTESIS@RISE, MEDCIDS, Faculty of Medicine of the University of Porto, Porto, Portugal

**Keywords:** pulmonary rehabilitation, women, STEM - science technology engineering mathematics, COPD - chronic obstructive pulmonary disease, cough, cognitive function, exercise training, home rehabilitation

**Editorial on the Research Topic** Women in science: Pulmonary rehabilitation

Worldwide, less than 30% of the workforce in research and development are women (1). Within the field of healthcare, however, female representation (74%) has made substantial gains (2). This is in line with data (2021) from the European Respiratory Society (ERS), showing a higher number of female members (69%) in comparison to male members within the “Allied Respiratory Professionals” Assembly, including amongst others lung function technologist, physiotherapists, nurses and psychologists. This is also visible in the Assembly's early career member category (less than 40 years of age), where women (68%) are also more represented. This increasing representativeness of the female gender is also seen in leadership roles, as five out of 10 (50%) most influenceable researchers (based on the number of publications in the last decade) in the field of pulmonary rehabilitation (PR) are female (3). The current topic on “Women in Science: Pulmonary Rehabilitation” celebrates the increasing representativeness and leadership of females in this research area. It intends to provide a stage for researchers who identify as a woman to present their research within the field of PR. In this topic collection, published research is clustered in two areas that can be considered hot topics in the latest years, namely regarding the settings where PR can be delivered and its role in the treatment of symptoms and extrapulmonary features.

## Pulmonary rehabilitation setting

Evidence of the effects of PR on people with chronic obstructive pulmonary disease (COPD) is unequivocal and its implementation is advocated in guidelines worldwide (4). Still, patients' access to and completion of PR is low, with 8% to 50% of patients referred to PR never attending, and from those who attend, 10% to 32% not completing it (5). The most common barriers to accessing PR include geographic distance, difficulty in commuting and disruption of daily life routine (6). Increasing the variety of settings in which PR is delivered has been suggested to improve accessibility (4), but research in this area is taking its first steps. This research topic includes a rapid review and a randomized controlled trial (RCT) exploring the effects of home-based and community-based PR programs on health outcomes in people with COPD.

In their rapid review, de Oliveira et al. highlighted the effects of home and community-based PR in improving exercise capacity and health-related quality of life of people with COPD. Nevertheless, its benefits in relation to other critical outcomes, such as physical activity are more controversial, possibly due to the paucity of evidence, heterogeneity of the interventions and incomplete reports on the programs' designs. The RCT from Horton et al. contributes to clarifying some of these issues by presenting the immediate effects of a well-structured home-based PR program in increasing patients' daily step count, above the minimal important difference (>1.100 steps) (7), and reducing sedentary time. Both studies emphasized the potential of home-based PR to increase access and completeness of PR by being safe, feasible, time-convenient, and flexible. In order to be confidently adopted into clinical practice, future guidelines which establish the safety and quality standards of these programs are needed.

## Non-pharmacological treatment of symptoms and extrapulmonary features

PR programs need to be comprehensive enough to properly manage symptoms other than dyspnoea and fatigue and also patients' extrapulmonary features. Chronic cough is one of the most prevalent symptoms in patients with chronic respiratory diseases (8) and cognitive dysfunction is a common comorbidity (9). Nevertheless, less attention has been paid to these patients' needs. This research topic, fortunately, included two research syntheses linked to these relevant topics, one systematic review focussed on the non-pharmacological management of non-productive chronic cough Ilicic et al. and one scoping review summarised the effects of exercise-based interventions on cognitive function in people with COPD Eastus et al.

Both reviews were based on a small number of studies (five in each) and participants (228 and 245), highlighting the recent interest of researchers in these topics. Non-pharmacological therapies for non-productive chronic cough included education, cough suppression, breathing techniques, mindfulness, and continuous positive airway pressure Ilicic et al. Exercise-based interventions were mostly integrated within PR programs Eastus et al. Although with different degrees of certainty, both reviews were able to show the beneficial effect of the studied intervention. The authors of the two reviews identified similar major limitations, the heterogeneity of study designs and of the outcomes used. To continuously improve these areas of research, authors recommended well-designed studies based on the most appropriate and validated outcomes. This is the only way forward to enable the development of guidelines informing on who should receive these adjuvant PR strategies and how these strategies should be delivered. From an academic point of view, these two reviews also highlighted the relevance of conducting research synthesis for the advancement of specific research areas.

## Gender differences in pulmonary rehabilitation

While not the focus of the included papers in this special topic, we like to take the opportunity to shortly address the presence of gender differences in chronic respiratory diseases and PR. Gender differences are critical to consider and incorporate into research and clinical care to optimise outcomes. Generally, women are more predisposed to develop bronchiectasis, pulmonary arterial hypertension and lymphangioleiomyomatosis, while men are predominantly affected by idiopathic pulmonary fibrosis (10). For more common respiratory diseases, e.g., COPD and asthma, there is a tendency for increased disease severity in women (10). For example, women with COPD seem to have more prevalent and severe traits, e.g., more frequent exacerbations and hospitalizations, activity-related dyspnoea, severe hyperinflation, reduced diffusion capacity, impaired mobility, symptoms of anxiety and depression, higher cardiovascular risk, and poor health status (11). The effectiveness of PR, however, does not seem to differ between males and females (12, 13), while non-attendance and non-adherence to PR seem predicted by the female gender (14, 15). Nevertheless, the current PR evidence seems, however, limited (12, 13).

## Concluding remarks

Within this special topic on “Women in Science: Pulmonary Rehabilitation” both original and synthesis research highlights highly relevant and pressing problems and knowledge gaps within the field. Two hot topics were addressed linked to PR delivery models, which are completely aligned with current concerns worldwide. For example, improving PR availability and access to PR was put forward as a recommendation within the recent Lancet Commission “Towards the elimination of COPD” (16). Next to providing new scientific insights, this research topic also has the purpose to provide attention to the scientists behind the research. All research included in this topic has been led by scientists who identify as a woman. While, the female representation in the field of PR is promising, we hope this research topic calls for action to further enhance and empower female careers in PR research. For example, by educating young woman to consider a career in science and that they can do it, and by providing successful female scientists a stage to act as role models. Additionally, this special topic on “Women in Science: Pulmonary Rehabilitation” also brings attention to gender differences in patients with chronic respiratory disease, with more research in the area of PR being highly warranted to provide optimal access, uptake and response to PR.
